# Integrated Network Pharmacology Analysis and Experimental Validation of Zadi‐5 Against Coronary Heart Disease

**DOI:** 10.1155/crp/5479556

**Published:** 2026-07-07

**Authors:** Jie Xiang, Jing Liu, Pengmei Liu

**Affiliations:** ^1^ Hygiene and Health Academy, Baotou Medical College, Baotou, China, btmc.cn

**Keywords:** coronary heart disease, network pharmacology, PPARG, Zadi-5

## Abstract

This study aimed to investigate the potential molecular mechanisms underlying the therapeutic effects of the traditional Mongolian medicine Zadi‐5 on coronary heart disease (CHD). The active compounds of Zadi‐5 were identified using the Traditional Chinese Medicine Systems Pharmacology Database and Analysis Platform, and their corresponding targets were retrieved from HERB. The GSE42148 dataset was used to analyze differentially expressed genes (DEGs) associated with CHD. A compound–target network and a disease–gene–target network were constructed, followed by Gene Ontology and Kyoto Encyclopedia of Genes and Genomes pathway enrichment analyses. Molecular docking was performed to predict binding modes. Furthermore, we employed a CHD model with high‐fat diet–fed ApoE^−/−^ mice to explore the protective effects of Zadi‐5 in CHD. A total of 250 targets related to the active compounds in Zadi‐5 were identified. Additionally, 480 DEGs were screened from the GSE42148 dataset, and seven key targets (MPO, IFNG, TOP2A, PTGS2, CYP2J2, F7, and PPARG) were identified. Molecular docking predicted strong binding affinities between PPARG and the main active components of Zadi‐5. Furthermore, Zadi‐5 markedly enhanced the heart performance and reduced fibrotic and inflammatory responses in vivo. Zadi‐5 effectively suppressed the levels of HIF‐1α and increased the PPARG levels in ApoE^−/−^ mice post‐myocardial infarction. These findings suggest that the active components of Zadi‐5 may exert therapeutic effects in CHD by targeting PPARG, as revealed by network pharmacology and molecular docking analyses.

## 1. Introduction

Coronary heart disease (CHD) is a cardiovascular disorder characterized by the narrowing or blockage of coronary arteries, leading to myocardial ischemia, hypoxia, or necrosis [1]. As the morbidity and mortality rates of CHD continue to increase globally, prevention and treatment have become critical priorities in modern medicine [2]. Currently, percutaneous coronary intervention (PCI) is the primary therapeutic approach for CHD, which aims to restore blood flow to the ischemic and hypoxic myocardium, thereby mitigating myocardial damage [3]. However, PCI is often associated with several limitations, including stent restenosis, distal microvascular obstruction, recurrent angina pectoris, persistent symptoms of blood stasis, and adverse cardiovascular events [4, 5].

The remarkable efficacy of traditional Chinese medicine (TCM) in treating CHD is well documented [6–8]. Among various traditional medical systems, Mongolian medicine has evolved by integrating the principles of TCM and Tibetan medicine [9]. It employs a treatment strategy for CHD based on syndrome differentiation and analgesia, emphasizing turbidity clearance to promote blood circulation, resolve blood stasis, and improve vascular function [10–12]. Zadi‐5, a traditional Mongolian medicine formula, is officially recognized by the classic Mongolian Medicine Chengfang Collection and the Drug Standard of the Ministry of Health of the People’s Republic of China (Volume of Mongolian Medicine) [13]. Zadi‐5 offers a unique multicomponent, multitarget approach for CHD treatment. Its formulation is designed to address multiple pathological mechanisms, including inflammation, fibrosis, and lipid metabolism dysregulation. This comprehensive strategy distinguishes Zadi‐5 from several conventional therapies that often focus on a single target, making it a promising candidate for the management of complex CHD cases. In recent years, network pharmacology, which integrates systems biology and multitarget pharmacology, has emerged as a powerful tool for elucidating the mechanisms of action of complex herbal formulations [14].

**FIGURE 1:**
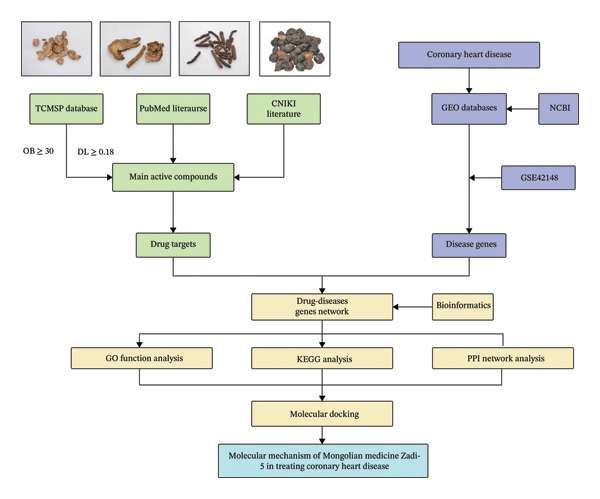
The mechanism of Zadi‐5 was elucidated with the experimental flowchart.

In this study, to explore the mechanism by which Zadi‐5 exerts its effects in the treatment of CHD, we selected “Rou Dou Kou” (*Myristica fragrans* Houtt), “Tu Mu Xiang” (*Inula helenium* L.), “Mu Xiang” (*Aucklandia lappa* Decne.), “Guang Zao” (*Choerospondias axillaris* Roxb. Burtt et Hill), and “Bi Ba” (*Piper longum* L.)—the main components of Zadi‐5—for network pharmacology analysis [15]. These components were analyzed to elucidate their potential synergistic mechanisms in the treatment of CHD. A workflow chart illustrating the mechanism of action of Zadi‐5 in CHD treatment is shown in Figure 1.

## 2. Materials and Methods

### 2.1. Screening of Active Components and Targets of Zadi‐5

Zadi‐5 (Inner Mongolia Mengyao Co. Ltd.; National Medicine Approval No. Z15020402) comprises “Rou Dou Kou” (*Myristica fragrans* Houtt), “Tu Mu Xiang” (*Inula helenium* L.), “Mu Xiang” (*Aucklandia lappa* Decne.), “Guang Zao” (*Choerospondias axillaris* Roxb. et Hill), and “Bi Ba” (*Piper longum* L.). All compounds from Rou Dou Kou, Mu Xiang, Guang Zao, and Bi Ba were analyzed using the Traditional Chinese Medicine Systems Pharmacology (TCMSP) database. The pharmacokinetic criteria for screening included an oral bioavailability greater than 30% and a drug‐likeness index exceeding 0.18.

Compound‐related targets were identified using the HERB online database (https://herb.ac.cn/). HERB was selected because it integrates target information from multiple authoritative sources, providing comprehensive coverage of TCM compound targets [16]. For each active compound, targets were retrieved from the HERB using both compound names and molecular structures as queries. Redundant targets were removed, and only targets with confidence scores ≥ medium were retained for subsequent analysis.

The UniProt protein database (https://www.uniprot.org/uploadlists/) was used to convert the identified target names into standardized gene nomenclature. All data used in this study were obtained from publicly available databases, and the analytical process fully complied with the usage guidelines of these databases.

### 2.2. Differentially Expressed Genes (DEGs) Identified in CHD

The dataset GSE42148 was downloaded from the Gene Expression Omnibus (GEO) database (https://www.ncbi.nlm.nih.gov/geo/). This dataset included gene expression profiles from 13 patients (aged 40–55 years) with angiographically confirmed coronary artery disease and 11 asymptomatic controls. Differential gene expression analysis was performed using the “limma” package in R. The criteria for selecting DEGs were set as a *p* value < 0.05 and an absolute fold change > 1.

### 2.3. Construction of Active Ingredient–Key Target Network

The relationship between the DEGs and the targets of action was visualized using a Venn diagram, which was generated using the online tool available at https://bioinformatics.psb.ugent.be/webtools/Venn/. Data pertaining to the active components and therapeutic targets of Zadi‐5 were collected, curated, and imported into Cytoscape (Version 3.7.1) to construct an active component–target interaction network centered on Zadi‐5. In this network, the nodes represent the active components and target proteins, whereas the edges denote the interactions between them. Subsequently, topological analysis was performed using the NetworkAnalyzer plugin to calculate three critical topological parameters, degree centrality, betweenness centrality, and closeness centrality, thereby facilitating the identification of the principal active components for subsequent mechanistic analysis. Topological parameters were calculated using Cytoscape’s NetworkAnalyzer with default settings: undirected graph analysis with unweighted edges, applying the Brandes algorithm for betweenness centrality and standard shortest path algorithms for closeness centrality calculations.

### 2.4. Gene Ontology (GO) and Kyoto Encyclopedia of Genes and Genomes (KEGG) Analysis

The overlapping targets identified from the Venn diagram analysis were uploaded to the INPUT database (https://cbcb.cdutcm.edu.cn/INPUT/) for GO enrichment analysis and to the DAVID database (https://david.ncifcrf.gov/) for KEGG pathway enrichment analysis. In GO enrichment analysis, three categories were considered: biological process (BP), molecular function (MF), and cellular component. These analyses aimed to elucidate the functional roles and pathways associated with the overlapping targets derived from Zadi‐5.

### 2.5. Molecular Docking

A molecular docking study was conducted to explore energetically favorable binding conformations of ligands within the active site of PPARG, as defined by Gupta et al. [17]. PPARG was selected for docking based on its highest network centrality metrics. The crystal structure of PPARG was downloaded from the RCSB PDB database (https://www1.rcsb.org/), and the pdb file was obtained. The mol2 files of the Zadi‐5 active ingredients were downloaded from the TCMSP database, and AutoDock Tools was used to process the protein crystals and compound structures. Finally, the files were converted into the PDBQT format, and molecular docking was conducted. The docking results were visualized using PyMOL software.

### 2.6. Establishment of the CHD Animal Model

All animal experiments were conducted in accordance with ARRIVE guidelines. The experimental procedures were approved by the Beijing Medconnor Laboratory Animal Welfare and Ethics Committee (approval no. MDKN‐2023‐278). Male mice lacking ApoE (ApoE^−/−^) purchased from Beijing Medconnor Laboratory, aged 6–8 weeks, underwent ligation of the left coronary artery’s anterior descending branch (LAD). Mice were anesthetized with isoflurane (Sigma‐Aldrich; 792632) inhalation in an induction chamber (induction: 4%–5%; maintenance: 1.5%–2%) mixed with 100% oxygen at a flow rate of 1 L/min. Anesthesia depth was assessed by the absence of the toe‐pinch withdrawal reflex. Under anesthesia, the mice were intubated with a small‐animal ventilator (Shanghai Yuyan Instruments) at a tidal volume of ∼200 μL and a respiratory rate of 120 breaths/min. The heart was exposed by separating the pericardium, and the LAD was ligated using an 8–0 nylon suture (Johnson & Johnson Ethicon; 1696G) below the left auricle. Sham‐operated male C57BL/6 mice (purchased from Beijing Medconnor Laboratory), aged 6–8 weeks, experienced the same procedure without LAD ligation. Subsequently, ApoE^−/−^ mice were assigned to three groups: the model group, the model + Zadi‐5‐L group (low dose), and the model + Zadi‐5‐H group (high dose). The low dose for human adults was 9 capsules thrice a day, and the high dose for human adults was 15 capsules thrice a day. Mice in the low‐ and high‐dose groups received Zadi‐5 at human‐equivalent (0.146 g) and double human‐equivalent (0.244 g) doses, respectively, for 4 weeks. The mice in the sham and model groups received an equivalent volume of water for the same duration.

### 2.7. Hematoxylin and Eosin (HE) and Masson’s Trichrome Staining

After the study, all animals were anesthetized by isoflurane inhalation (1.5%–2%, Sigma‐Aldrich; 792632) and euthanized by cervical dislocation. Samples were preserved using a 4% phosphate‐buffered formaldehyde solution at a pH of 7.4, followed by a gradual dehydration process. They were then embedded in paraffin and transversely sectioned to a uniform thickness. Sections were stained with HE using HE staining kit (Beyotime; C0105S), adhering to the instructions provided by the supplier. To evaluate myocardial inflammatory cell infiltration, a random selection of 10 microscopic fields at 400× magnification within the area of myocardial infarction in each heart specimen was examined using a microscope (BX53 + DP72; Olympus, Japan). As for Masson’s trichrome staining, cardiac tissues were encased in paraffin and sectioned into 5‐μm‐thick slices, which were subsequently processed with Masson’s trichrome staining following the manufacturer’s guidelines (Beyotime; C0189S). To assess infarct fibrosis, measurements were obtained from the base, midpapillary, and apex of the hearts, all of which were stained with Masson’s trichrome and viewed under an Olympus microscope. The cardiac fibrosis area was quantified using ImageJ software.

### 2.8. Immunohistochemistry Staining

For immunohistochemical analysis, antigen retrieval was achieved through the high‐pressure boiling method with sodium citrate buffer following deparaffinization. After three rinses with PBS, nonspecific binding was blocked with bovine serum albumin (Sigma‐Aldrich; A2153) for 45 min. Sections were then incubated with primary antibodies specific to HIF‐1α (ab228649, 1/100, Abcam) or PPARG (ab272718, 1/100, Abcam) at 4°C overnight, followed by treatment with a secondary antibody conjugated to horseradish peroxidase (ab205718, 1/2000, Abcam). After another three washes with PBS, the cell nuclei were counterstained with hematoxylin for 3 min at room temperature. The stained sections were visualized under a microscope, and the quantification of positive staining areas was performed using ImageJ software.

### 2.9. Statistical Analysis

Results were presented as mean ± standard error of the mean, with each experiment in this research being conducted a minimum of three times independently. Data analysis was performed using SPSS Statistics Version 26. One‐way ANOVA was used to assess statistical significance across multiple groups. Statistical significance was set at *p* < 0.05.

## 3. Results

### 3.1. Screening for Active Compounds

Based on the analysis of the TCMSP database, a total of 40 active compounds were identified in the ingredients of Zadi‐5. Specifically, 9 active compounds were derived from “Rou Dou Kou” (*Myristica fragrans* Houtt), another 9 from “Mu Xiang” (*Aucklandia lappa* Decne.), 2 from “Guang Zao” (*Choerospondias axillaris* Roxb. et Hill), and 16 from “Bi Ba” (*Piper longum* L.). The detailed results of the active compound identification are summarized in Table 1.

**TABLE 1:** The active compounds identified from Rou Dou Kou (Myristicae Semen), Mu Xiang (Aucklandiae Radix), Guang Zao (Choerospondiatis Fructus), and “Bi Ba” (Piperis Longi Fructus).

Drug	Mol ID	Molecule name	OB (%)	DL
Myristicae Semen	MOL000358	Beta‐sitosterol	36.91	0.75
Myristicae Semen	MOL009254	Galbacin	61	0.53
Myristicae Semen	MOL009255	5‐[(2S,3S)‐7‐Methoxy‐3‐methyl‐5‐[(E)‐prop‐1‐enyl]‐2,3‐dihydrobenzofuran‐2‐yl]‐1,3‐benzodioxole	53.11	0.4
Myristicae Semen	MOL009259	Kudos	45.06	0.38
Myristicae Semen	MOL009264	Tetrahydrofuroguaiacin B	62.86	0.32
Myristicae Semen	MOL009265	Threo‐austrobailignan‐5	49.49	0.32
Myristicae Semen	MOL009263	Saucernetindiol	41.85	0.32
Myristicae Semen	MOL009243	Isoguaiacin	48.78	0.31
Myristicae Semen	MOL007920	meso‐1,4‐Bis‐(4‐hydroxy‐3‐methoxyphenyl)‐2,3‐dimethylbutane	31.32	0.26
Aucklandiae Radix	MOL000211	Mairin	55.38	0.78
Aucklandiae Radix	MOL000449	Stigmasterol	43.83	0.76
Aucklandiae Radix	MOL000359	Sitosterol	36.91	0.75
Aucklandiae Radix	MOL010839	Lappadilactone	38.56	0.73
Aucklandiae Radix	MOL010828	Cynaropicrin	67.5	0.38
Aucklandiae Radix	MOL010813	Benzo[a]carbazole	35.22	0.22
Choerospondiatis Fructus	MOL000358	Beta‐sitosterol	36.91	0.75
Choerospondiatis Fructus	MOL001002	Ellagic acid	43.06	0.43
Choerospondiatis Fructus	MOL001490	Bis [(2S)‐2‐ethylhexyl] benzene‐1,2‐dicarboxylate	43.59	0.35
Choerospondiatis Fructus	MOL000098	Quercetin	46.43	0.28
Choerospondiatis Fructus	MOL001736	(−)‐Taxifolin	60.51	0.27
Choerospondiatis Fructus	MOL000096	(−)‐Catechin	49.68	0.24
Choerospondiatis Fructus	MOL000422	Kaempferol	41.88	0.24
Choerospondiatis Fructus	MOL004328	Naringenin	59.29	0.21
Choerospondiatis Fructus	MOL001040	(2R)‐5,7‐Dihydroxy‐2‐(4‐hydroxyphenyl)chroman‐4‐one	42.36	0.21
Piperis Longi Fructus	MOL001558	Sesamin	56.55	0.83
Piperis Longi Fructus	MOL001607	ZINC03982454	36.91	0.76
Piperis Longi Fructus	MOL001594	Pisatin	88.05	0.64
Piperis Longi Fructus	MOL001555	ZINC03996196	52.35	0.62
Piperis Longi Fructus	MOL001610	Sylvatine	44	0.51
Piperis Longi Fructus	MOL001614	(E,E,E)‐11‐(1,3‐Benzodioxol‐5‐yl)‐N‐(2‐methylpropyl)‐2,4,10‐undecatrienenamide	42.72	0.43
Piperis Longi Fructus	MOL001560	Pipernonaline	51.32	0.41
Piperis Longi Fructus	MOL001561	Dehydropipernonaline	47.73	0.41
Piperis Longi Fructus	MOL001616	1‐[1‐Oxo‐9 (3, 4‐methylenedioxyphenyl)‐2E,8E‐nonadienyl] pyrrolidine	49.43	0.36
Piperis Longi Fructus	MOL001601	1,2,5,6‐Tetrahydrotanshinone	38.75	0.36
Piperis Longi Fructus	MOL001588	N‐Isobutyleicosa‐2 (E),4 (E),8 (Z)‐trienamide	44.48	0.32
Piperis Longi Fructus	MOL001589	N‐Isobutyl‐2,4‐icosadienamide	38.86	0.32
Piperis Longi Fructus	MOL001592	Piperine	42.52	0.23
Piperis Longi Fructus	MOL001586	N‐(2,5‐Dimethoxyphenyl)‐4‐methoxybenzamide	60.7	0.18
Piperis Longi Fructus	MOL001621	l‐Undecylenyl‐3,4‐methylenedioxybenzene	47.97	0.18
Piperis Longi Fructus	MOL001559	Piperlonguminine	30.71	0.18

Abbreviations: DL, drug‐likeness; OB, oral bioavailability.

### 3.2. Screening for Key Targets in CHD

**FIGURE 2:**
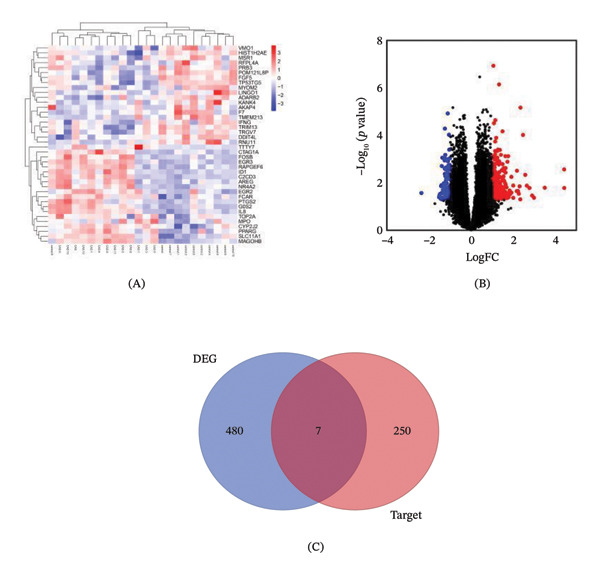
Screening for key targets in CHD. The DEGs in CHD were expressed as heatmap (A) and volcano map (B). (C) Venn diagram for 250 compound‐related targets obtained from HERB database and 480 DEGs.

A total of 48/XMLSchema Schema documents could not be parsed. A total of 480 DEGs were identified, of which 347 were downregulated and 141 were upregulated (Figure 2A, B). To identify key targets relevant to both Zadi‐5 compounds and CHD, we performed a Venn diagram analysis comparing 250 compound‐related targets with the 480 DEGs. This analysis revealed seven key targets: MPO, IFNG, TOP2A, PTGS2, CYP2J2, F7, and PPARG (Table 2).

**TABLE 2:** The seven key targets in CHD.

Intersection gene	LogFC	*p* value
PTGS2	1.5552378	1.78E − 02
PPARG	1.4910769	1.34E − 03
TOP2A	1.171035	3.80E − 02
CYP2J2	1.0752378	6.27E − 04
MPO	1.056972	2.06E − 02
IFNG	−1.1015521	7.94E − 03
F7	−1.2905734	1.85E − 02

### 3.3. Functional Pathway Analysis of Key Targets

**FIGURE 3:**
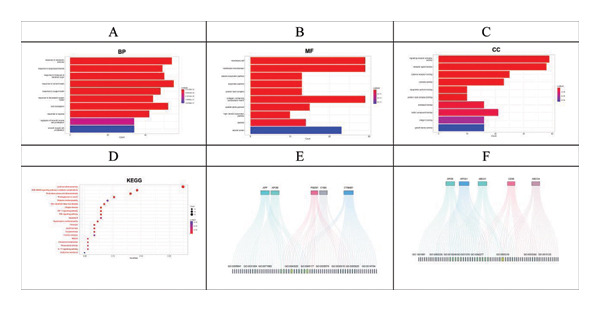
Functional pathway analysis of DEGs in CHD. (A, E) Biological process, (B, F) molecular function, and (C) cellular component of GO enrichment analysis. (D) KEGG analysis of DEGs in CHD.

The 480 DEGs identified in CHD were uploaded to the INPUT database for GO enrichment analysis and to the DAVID database for KEGG pathway analysis. GO enrichment analysis revealed that, according to BP analysis, the DEGs are involved in response to xenobiotic stimuli, nutrient levels, and lipid localization (Figure 3A). MF analysis indicated that these genes may regulate membrane rafts, membrane microdomains, and collagen‐containing extracellular matrix components (Figure 3B). Furthermore, signaling receptor activator and receptor ligand activities emerged as potential focal points for CHD treatment with Zadi‐5 (Figure 3C). KEGG pathway analysis demonstrated that the DEGs were enriched in pathways related to lipid metabolism and atherosclerosis, AGE‐RAGE signaling in diabetic complications, and fluid shear stress and atherosclerosis (Figure 3D).

### 3.4. TCM–Active Ingredient–Key Target Network

**FIGURE 4:**
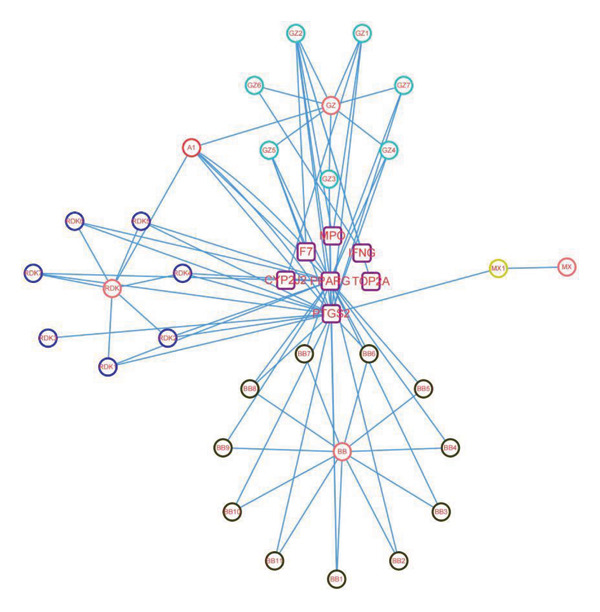
TCM–active ingredient–key target network of Zadi‐5 and CHD. The network consists of 38 nodes, including 4 herbal components of Zadi‐5, 27 active compounds, and 7 key targets. Specifically, there are 8 active ingredients in Rou Dou Kou (RDK1, RDK2, RDK3, RDK4, RDK5, RDK6, RDK7, and A1), 11 active ingredients in Bi Ba (BB1, BB2, BB3, BB4, BB5, BB6, BB7, BB8, BB9, BB10, and BB11), 8 active ingredients in Guang Zao (GZ1, GZ2, GZ3, GZ4, GZ5, GZ6, GZ7, and A1), and 1 active ingredient in Mu Xiang (MX1). Among them, Rou Dou Kou and Guang Zao share a common active ingredient (A1). Key nodes such as MPO, IFNG, TOP2A, PTGS2, CYP2J2, and F7 play central roles in the network. The edges represent interactions between compounds and targets, illustrating the multitarget and multipathway mechanisms of Zadi‐5 in CHD treatment.

The TCM–active ingredient–key target network was constructed using Cytoscape 3.7.1 software and is depicted in Figure 4. The compound–target network illustrates the complex interactions between the active components of Zadi‐5 and their corresponding targets. This network comprises 38 nodes, including 4 TCM, 27 chemical components, and 7 key targets. Specifically, there were 8 active ingredients in Rou Dou Kou (RDK1, RDK2, RDK3, RDK4, RDK5, RDK6, RDK7, and A1), 11 active ingredients in Bi Ba (BB1, BB2, BB3, BB4, BB5, BB6, BB7, BB8, BB9, BB10, and BB11), 8 active ingredients in Guang Zao (GZ1, GZ2, GZ3, GZ4, GZ5, GZ6, GZ7, and A1), and 1 active ingredient in Mu Xiang (MX1). Among these, Rou Dou Kou and Guang Zao share a common active ingredient (A1). Key nodes, such as MPO, IFNG, TOP2A, PTGS2, CYP2J2, and F7, play central roles in the network, reflecting their importance in regulating lipid metabolism, inflammation, and fibrosis. This network highlights the multitarget and multipathway mechanisms of Zadi‐5 and provides insights into its potential therapeutic effects in CHD. PPARG was identified as a crucial target for the active ingredients present in Zadi‐5.

### 3.5. Molecular Docking

**FIGURE 5:**
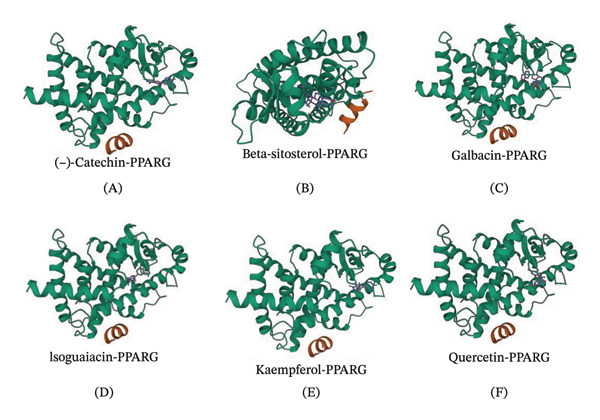
Molecular docking between active ingredient and PPARG. (A) Catechin, (B) beta‐sitosterol, (C) galbacin, (D) isoguaiacin, (E) kaempferol, and (F) quercetin docking with PPARG.

PPARG, identified as the key target of the active ingredients of Zadi‐5, was selected for molecular docking studies. The top six active ingredients were selected based on their potential interactions and docked to PPARG using AutoDock Vina. The molecular docking results indicated that catechin, beta‐sitosterol, galbacin, isoguaiacin, kaempferol, and quercetin could effectively bind to PPARG (Figure 5A–F). The docking scores are presented in Table 3. Specifically, catechin (affinity: −7.796 kcal/mol), beta‐sitosterol (affinity: −7.589 kcal/mol), and galbacin (affinity: −9.009 kcal/mol) exhibited the strongest binding affinities. Furthermore, specific binding patterns between PPARG and the six compounds were visualized and optimized using PyMOL 2.3.0.

**TABLE 3:** The top six active ingredients docked to PPARG and the corresponding docking scores.

Drug–protein	Binding energy (Kcal/mol)
Beta‐sitosterol–PPARG	−7.589
Galbacin–PPARG	−9.009
Isoguaiacin–PPARG	−6.884
Quercetin–PPARG	−7.473
Kaempferol–PPARG	−7.307
(−)‐Catechin–PPARG	−7.796

### 3.6. Zadi‐5 Attenuated Myocardial Inflammation and Fibrosis In Vivo

**FIGURE 6:**
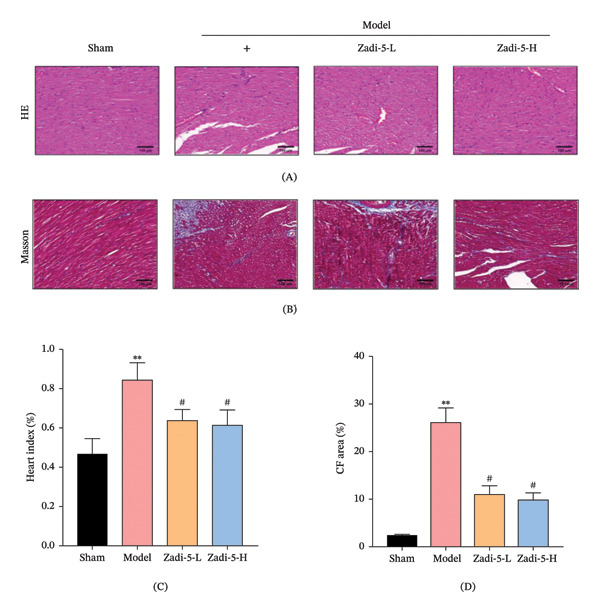
Zadi‐5 attenuated cardiac fibrosis and inflammation in CHD model mice (*n* = 6). Representative images of (A) HE staining and (B) Masson trichrome staining of cardiac tissues. (C) Statistical results of heart index. (D) Statistical results of cardiac fibrosis. ^∗∗^ *p* < 0.01 (vs. sham group); ^#^ *p* < 0.05 (vs. model group).

**FIGURE 7:**
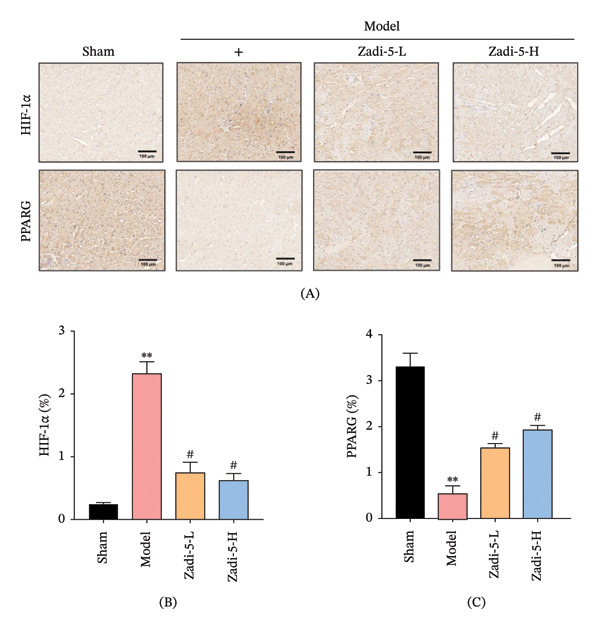
Zadi‐5 decreased expression of HIF‐1α and increased PPARG (*n* = 6). (A) Representative images of immunohistochemical analysis of cardiac tissues. (B) Statistical results of expression of HIF‐1α. (C) Statistical results of expression of PPARG. ^∗∗^ *p* < 0.01 (vs. sham group); ^∗^ *p* < 0.05 (vs. model group).

Subsequently, we investigated whether the therapeutic effect of Zadi‐5 in the CHD model mice is related to PPARG. The cardioprotective effects of Zadi‐5 were further validated by histopathological analysis using HE and Masson staining on cardiac tissue sections. HE staining revealed extensive inflammatory cell infiltration and myocardial structural disruption in the model group, which was mitigated by the administration of low and high doses of Zadi‐5, suggesting its anti‐inflammatory action in cardiac protection (Figure 6A). Masson’s trichrome staining highlighted substantial collagen accumulation in the model group, and treatment with both doses of Zadi‐5 significantly decreased the extent of myocardial fibrosis (Figure 6B, D). The model group exhibited a higher heart‐to‐body weight ratio than the sham group, which was significantly reduced by both low and high doses of Zadi‐5 (Figure 6B). Moreover, utilizing network pharmacology and molecular docking, HIF‐1α and PPARG were identified as potentially crucial. To elucidate how Zadi‐5 mitigates cardiac injury, we conducted immunohistochemistry assays to evaluate the protein levels of HIF‐1α and PPARG. In contrast to the sham group, there was an upregulation of HIF‐1α and a downregulation of PPARG in the model group, a pattern that was reversed with both low and high doses of Zadi‐5 (Figure 7A–C).

## 4. Discussion

In this study, we investigated the therapeutic potential of the Mongolian medicine Zadi‐5 for CHD. Using a network pharmacology approach, we systematically collected and analyzed research data from multiple databases to elucidate the mechanisms of action of Zadi‐5, leveraging the multicomponent, multitarget, and multipathway characteristics typical of TCM compounds. By constructing and enriching the Zadi‐5 component–target–disease network, we identified potential targets and mechanisms of action for its active ingredients. Molecular docking studies were conducted to predict the interactions between the drug components and the disease‐related protein molecule PPARG. Zadi‐5 downregulated PPARG levels and attenuated myocardial inflammation and fibrosis in vivo. Based on our findings, future research involving cell model experiments and animal studies is warranted to validate whether Zadi‐5 exerts its therapeutic effects on CHD through the PPARG pathway. This study provides a foundational framework for understanding the pharmacological basis of Zadi‐5 in CHD treatment and highlights its potential as a multitarget therapeutic agent.

Compared to modern medicines, such as statins, antiplatelet agents, and beta‐blockers, Zadi‐5 offers a unique advantage by targeting multiple pathways involved in CHD pathogenesis. Zadi‐5 is composed of five medicinal components: Rou Dou Kou (*Myristica fragrans* Houtt), Tu Mu Xiang (*Inula helenium* L.), Mu Xiang (*Aucklandia lappa* Decne.), Guang Zao (*Choerospondias axillaris* Roxb. Burtt & Hill), and Bi Ba (*Piper longum* L.). Among these, Rou Dou Kou is one of the most frequently used herbal pairs in traditional Mongolian medicine to treat CHD [11, 18]. Additionally, Tu Mu Xiang, Mu Xiang, and Guang Zao have been reported to alleviate CHD through various mechanisms [11, 13, 18]. Therefore, identifying the active ingredients and target genes of Zadi‐5 is crucial for understanding its therapeutic potential in CHD treatment.

Based on the network analysis and molecular docking results, the primary components of Zadi‐5 appear to exert preventive and therapeutic effects on CHD by modulating key CHD‐related genes, including MPO, IFNG, TOP2A, PTGS2, CYP2J2, F7, and PPARG. For instance, MPO, an enzyme with a well‐established prognostic role in coronary artery disease, has emerged as a promising biomarker for cardiac risk stratification [19]. Wang et al. further demonstrated its involvement in the drug–gene network of coronary artery disease through bioinformatic analysis [20]. Similarly, aberrant expression of the IFNG gene has been implicated as a potential risk factor in coronary artery disease [21]. The expression of PTGS2 is positively correlated with the severity of atherosclerosis [22] and has been identified as a hub gene for the diagnosis of coronary artery disease [23]. A frequent promoter polymorphism in CYP2J2 has been associated with reduced gene expression and an increased risk of coronary artery disease [24, 25]. Additionally, the F7 gene has been linked to an elevated CHD risk [26]. The identification of PPARG as a key target for Zadi‐5 aligns with its well‐established role in the regulation of lipid metabolism and inflammation [27–29], which are central to CHD pathogenesis [30, 31]. However, the effects of PPARG activation can vary depending on the clinical context. For example, although some studies have demonstrated that PPARG activation improves lipid profiles and reduces inflammation [32, 33], others have reported neutral or adverse effects in patients with advanced atherosclerosis or specific comorbidities [34]. Additionally, genetic polymorphisms such as Pro12Ala have been associated with varying effects on CHD risk across different populations, suggesting that the therapeutic potential of PPARG may be influenced by individual genetic and environmental factors [35]. These findings underscore the need for further research to clarify the role of PPARG in CHD and to identify patient subgroups that may benefit the most from PPARG‐targeted therapies.

Our study suggests that PPARG can bind to the selected active ingredients of Zadi‐5, indicating its potential as a target gene for Zadi‐5 in CHD treatment. This finding is consistent with those of previous studies. González‐Castro et al. proposed that PPARG may serve as a biomarker of CHD risk [36]. However, other studies have highlighted its association with CHD [37, 38]. Furthermore, molecular docking experiments with the top six active ingredients of Zadi‐5—catechin, beta‐sitosterol, galbacin, isoguaiacin, kaempferol, and quercetin—revealed their ability to bind to PPARG. These results suggest that Zadi‐5 may exert its therapeutic effects on CHD by modulating the PPARG gene. Future studies should also extend docking to additional hub targets to comprehensively characterize the multitarget binding landscape.

Additionally, the seven identified target genes were mainly enriched in pathways related to lipid metabolism and atherosclerosis, AGE‐RAGE signaling in diabetic complications, and fluid shear stress and atherosclerosis. Atherosclerosis contributes to clinical manifestations, such as CHD, ischemic stroke, and peripheral vascular disease, by narrowing the lumen or through thrombus formation that impedes blood flow. Among these, CHD is the most prevalent and encompasses conditions such as stable angina pectoris and acute coronary syndrome [39]. The KEGG pathway analysis revealed that the key targets of Zadi‐5 are enriched in pathways related to lipid metabolism, atherosclerosis, and inflammation, which are central to CHD pathogenesis. These findings suggest that Zadi‐5 may exert its therapeutic effects by modulating these pathways, offering a multitarget approach for CHD treatment. In particular, the regulation of PPARG and lipid metabolism pathways aligns with the mechanisms of statins, a cornerstone of CHD therapy. Therefore, we hypothesized that Zadi‐5 exerts its therapeutic effects on CHD by regulating PPARG through pathways associated with lipid metabolism and atherosclerosis. Specifically, our findings suggested that the active components of Zadi‐5 could inhibit CHD progression by modulating these pathways. However, unlike statins, Zadi‐5’s multicomponent nature may provide additional benefits for addressing inflammation and fibrosis, which are often inadequately managed by conventional treatments. The results of in vivo animal experiments also indicated that Zadi‐5 attenuated myocardial inflammation and fibrosis.

Notably, this study identified CHD‐related targets based on DEGs from a single GEO dataset. Future studies should integrate multidatabase mining at the initial screening stage to construct a more comprehensive disease target repertoire and enhance the robustness of the network construction.

In conclusion, this study is the first attempt to use network pharmacology to elucidate the molecular mechanisms underlying the therapeutic effects of Zadi‐5 on CHD. Our results indicate that the effective components of Zadi‐5 may mitigate the onset and development of CHD by targeting PPARG and influencing pathways related to lipid metabolism and atherosclerosis. These insights provide a foundation for future experimental validation and potential therapeutic applications.

## Author Contributions

P.L. conceived the study; J.X. and J.L. conducted the experiments; P.L. analyzed the data; and J.X. and J.L. were major contributors in writing the manuscript.

## Funding

This study was supported by Baotou Medical College Research Fund Project Youth Program (BYJJ‐ZRQM 202211).

## Disclosure

All authors have read and approved the final manuscript. All authors agree to be accountable for the content and conclusions of the article.

## Ethics Statement

This study was approved by the Ethics Committee of Beijing Medconnor Laboratory Animal Welfare and Ethics Committee (approval no. MDKN‐2023‐278). All experiments were performed in accordance with relevant guidelines and regulations.

## Conflicts of Interest

The authors declare no conflicts of interest.

## Supporting Information

Additional supporting information can be found online in the Supporting Information section.

## Supporting information


**Supporting Information 1** Supporting File 1. Full study protocol approved by the ethics committee.docx.

**Supporting Information 2** Supporting File 2. Purchase orders.docx.

**Supporting Information 3** Supporting File 3. Raw data.rar.

## Data Availability

The datasets used and/or analyzed during the current study are available from the corresponding author on reasonable request.
