# Supplementary material for: Integrated Network Pharmacology Analysis and Experimental Validation of Zadi‐5 Against Coronary Heart Disease
Source: Cardiol Res Pract. 2026 Jul 7;2026:5479556. doi: 10.1155/crp/5479556 (PMC13338708; doi:10.1155/crp/5479556)
Supplement: Supplementary file 1 — Supporting Information 1 Supporting File 1. Full study protocol approved by the ethics committee.docx. [file CRP-2026-5479556-s001.docx]

The complete study protocol, titled "**Molecular Mechanism of Traditional Mongolian Medicine Zadi-5 Against Coronary Heart Disease Based on Network Pharmacology**" (Project No. MDKN-2023-278), was submitted to and approved by the Beijing Medconnor Laboratory Animal Welfare and Ethics Committee on June 10, 2023. The principal investigator was Dr. Xiang Jie (Baotou Medical College, School of Health). The anticipated project duration was from June 2023 to June 2024. No external funding was received for this study.

**Study Objective:** To investigate the therapeutic effects and underlying molecular mechanisms of Zadi-5, a traditional Mongolian medicine formulation, in coronary heart disease (CHD) using an integrated network pharmacology approach combined with in vivo experimental validation.

**Animal Information and Housing:** A total of 34 male mice were used in this study, including 24 ApoE-deficient (ApoE^−/−^) mice and 10 C57BL/6 wild-type mice, all aged 6–8 weeks. The animals were housed under standard laboratory conditions with a 12-hour light/dark cycle, controlled temperature (22 ± 2°C), and free access to food and water. All experimental procedures were conducted in accordance with the ARRIVE guidelines.

**Experimental Design and Grouping:** The CHD model was established by ligation of the left anterior descending coronary artery (LAD) in ApoE^−/−^ mice. Briefly, mice were anesthetized and intubated with a small-animal ventilator (39 mL/min). The heart was exposed via a left thoracotomy, and the LAD was ligated with an 8–0 nylon suture below the left auricle. Sham-operated C57BL/6 mice underwent the identical surgical procedure without LAD ligation.

Following surgery, ApoE^−/−^ mice were randomly allocated into three groups (n = 6 per group):

Model group: ApoE^−/−^ mice with LAD ligation, receiving distilled water by gavage for 4 weeks.

Model + Zadi-5-L group: ApoE^−/−^ mice with LAD ligation, receiving Zadi-5 at a low dose (human-equivalent dose of 0.146 g) by gavage for 4 weeks.

Model + Zadi-5-H group: ApoE^−/−^ mice with LAD ligation, receiving Zadi-5 at a high dose (double human-equivalent dose of 0.244 g) by gavage for 4 weeks.

The sham-operated C57BL/6 mice (n = 10, with 6 used for histological and molecular analyses) received an equivalent volume of distilled water for the same duration. The human-equivalent doses were calculated based on the clinical adult dosage (low dose: 9 capsules three times daily; high dose: 15 capsules three times daily).

**Endpoint Assessments and Outcome Measures:** The primary endpoints included (1) cardiac function and morphology, (2) myocardial inflammation and fibrosis, and (3) protein expression of key targets. Specifically:

**General observations:** Body weight and heart-to-body weight ratio were recorded at the end of the 4-week treatment period.

**Histopathological analysis:** Cardiac tissues were collected and fixed in 4% phosphate-buffered formaldehyde (pH 7.4). Paraffin-embedded sections (5 μm) were subjected to hematoxylin-eosin (HE) staining to evaluate inflammatory cell infiltration and myocardial structural integrity, and Masson's trichrome staining to quantify collagen deposition and fibrotic area. Ten random microscopic fields (400× magnification) within the infarcted region were examined per heart.

Immunohistochemistry (IHC): Paraffin sections were processed for antigen retrieval and incubated with primary antibodies against HIF-1α (ab228649, 1:100, Abcam) and PPARG (ab272718, 1:100, Abcam) overnight at 4°C. After secondary antibody incubation and hematoxylin counterstaining, positive staining areas were quantified using ImageJ software.

**Network pharmacology and molecular docking:** Active compounds of Zadi-5 were screened from the TCMSP database (oral bioavailability > 30%, drug-likeness > 0.18). Targets were retrieved from the HERB database, and differentially expressed genes (DEGs) were identified from the GEO dataset GSE42148. GO and KEGG enrichment analyses were performed using the INPUT and DAVID databases, respectively. Molecular docking of top active ingredients to PPARG was conducted using AutoDock Vina.

**Humane Endpoints and Euthanasia:** Mice were monitored daily for signs of distress, including severe lethargy, labored breathing, or body weight loss exceeding 20%. No animals reached the predetermined humane endpoints prior to the scheduled sacrifice. At the end of the study, all animals were deeply anesthetized by isoflurane inhalation (1.5–2%) and euthanized by cervical dislocation. Cardiac tissues were immediately harvested for subsequent analyses. The approved protocol also included overdose of anesthetics as an alternative euthanasia method.

**Sample Size Justification:** The sample size of n = 6 per group was determined based on our preliminary data and published literature using similar CHD animal models, which indicated that this number would provide sufficient statistical power (≥ 80%) to detect a 25% difference in myocardial fibrosis area with an alpha level of 0.05.
