# Supplementary material for: Integrated Network Pharmacology Analysis and Experimental Validation of Zadi‐5 Against Coronary Heart Disease
Source: Cardiol Res Pract. 2026 Jul 7;2026:5479556. doi: 10.1155/crp/5479556 (PMC13338708; doi:10.1155/crp/5479556)
Supplement: Supplementary file 2 — Supporting Information 2 Supporting File 2. Purchase orders.docx. [file CRP-2026-5479556-s002.docx]

| **Material Name** | **Specification/Strain** | **Common Manufacturer & Catalog No. (Reference)** |
| --- | --- | --- |
| ApoE knockout mice | Male, 6–8 weeks old | Beijing Medconnor |
| C57BL/6 mice | Male, 6–8 weeks old | Beijing Medconnor |
| Zadi-5 | Low dose: 0.146 g/mouse/day; High dose: 0.244 g/mouse/day | Inner Mongolia Mengyao Co., Ltd.; National Medicine Approval No. Z15020402 |
| Isoflurane | Inhalation anesthetic | Sigma-Aldrich 792632 |
| 8-0 Nylon suture | 8-0 | Johnson & Johnson Ethicon 1696G |
| Small-animal ventilator and accessories | Ventilation rate: 39 mL/min | Shanghai Yuyan Instruments |
| HE staining kit |  | Beyotime C0105S |
| Masson's trichrome staining kit |  | Beyotime C0189S |
| Rabbit anti-HIF-1α primary antibody |  | Abcam ab228649 |
| Rabbit anti-PPARG primary antibody |  | Abcam ab272718 |
| HRP-conjugated secondary antibody |  | Abcam ab205718 (Goat Anti-Rabbit IgG H&L HRP) |
| Bovine serum albumin |  | Sigma-Aldrich A2153 |
| Upright microscope and imaging system |  | Olympus BX53+DP72, Japan |
